# Computed tomography multi-phase radiomics models for differentiating benign and malignant thyroid nodules

**DOI:** 10.3389/fendo.2026.1928263

**Published:** 2026-10-15

**Authors:** Ning Li, Jianan Tong, Guozheng Zhang, Shufeng Xu, Weitao Huang, Xiaowei Han, Jingfeng Zhang, Xisong Zhu

**Affiliations:** 1 Jinhua Graduate Joint Training Base, Zhejiang Chinese Medical University, Jinhua, China; 2 Department of Radiology, Wenzhou Medical University Affiliated Quzhou Hospital (Quzhou People’s Hospital), Quzhou, China; 3 Department of Quzhou Key Laboratory of Respiratory Mechanics in Critical Care Medicine, Wenzhou Medical University Affiliated Quzhou Hospital (Quzhou People’s Hospital), Quzhou, China; 4 Department of Radiology, Ningbo No. 2 Hospital, Ningbo, China

**Keywords:** computed tomography, differential diagnosis, multiphase enhancement, preoperative risk stratification, radiomics, thyroid nodules

## Abstract

**Objective:**

To develop and validate an integrated model combining multiphase CT radiomics features, clinical data, and conventional morphological CT signs for the preoperative differentiation of benign and malignant thyroid nodules.

**Methods:**

We retrospectively enrolled patients with surgically confirmed thyroid nodules who underwent preoperative contrast-enhanced thyroid CT examination at two centers. Patients from center 1 were randomly allocated to a training cohort and an internal validation cohort at an 8:2 ratio; patients from center 2 served as an external validation cohort. Clinical characteristics and morphological features of nodules were assessed. Radiomics features were extracted from all three CT phases, and four categories of models were constructed: clinical model, single-phase CT radiomics models, multiphase CT radiomics model, and combined clinical-radiomics model. Model performance and interpretability were evaluated using receiver operating characteristic (ROC) curve analysis, calibration curves, decision curve analysis (DCA), and SHapley Additive exPlanations (SHAP).

**Results:**

The multiphase CT radiomics model yielded areas under the ROC curve (AUCs) of 0.919, 0.836, and 0.787 in the training, internal validation, and external validation cohorts, respectively. Among the single-phase models, the venous-phase model achieved the best external validation performance (AUC = 0.815). The combined model, integrating clinical and morphological features with multiphase radiomics features, achieved the highest overall diagnostic performance, with an external validation AUC of 0.865 (sensitivity = 74.0%, specificity = 84.5%), significantly outperforming both the clinical model (AUC = 0.800) and the multiphase radiomics model (AUC = 0.787). It also demonstrated favorable calibration and greater clinical net benefit.

**Conclusion:**

Multiphase CT radiomics enables effective differentiation of benign and malignant thyroid nodules. Integration of clinical and morphological information further improves diagnostic performance, providing an objective, noninvasive tool for preoperative risk stratification of incidentally detected thyroid nodules on CT.

## 1 Introduction

The detection rate of thyroid nodules continues to rise globally, with ultrasound identifying nodules in approximately 65% of the general population, 5% to 15% of which are malignant (1–3). Thyroid cancer is the most prevalent endocrine malignancy, with a higher incidence in females; however, male patients tend to exhibit more aggressive tumor biology (4–6). While most thyroid cancers follow an indolent clinical course, a subset may develop regional lymph node metastasis, extrathyroidal extension, or compressive symptoms (7). Accurate preoperative characterization of thyroid nodules is therefore critical to guide clinical decision-making, avoid overtreatment, and improve patient prognosis (8).

Ultrasound is the first-line imaging modality for thyroid nodule evaluation and allows real-time guidance for fine-needle aspiration biopsy (FNAB). Nevertheless, its diagnostic reliability is constrained by operator dependence, imaging artifacts, and sampling error (9–11). FNAB, although widely used as a reference standard, is also limited by inadequate specimen acquisition and overlapping cytological features (12). Computed tomography (CT) offers excellent spatial resolution and soft-tissue contrast, enabling detailed delineation of the relationship between nodules and the thyroid capsule, trachea, adjacent vessels, and cervical lymph nodes, thus serving as a valuable complement to ultrasound (5, 13).

Radiomics allows high-throughput extraction of quantitative imaging features that objectively capture the underlying heterogeneity of lesions (14). A recent systematic review and meta-analysis pooling 12 CT-based radiomics studies reported a pooled area under the curve (AUC) of 0.894 (sensitivity = 0.85, specificity = 0.83) for differentiating malignant from benign thyroid nodules, and demonstrated that radiomics models significantly outperformed radiologists’ visual assessment (AUC: 0.853 vs. 0.770, p < 0.001) (15). However, most individual studies are limited by small sample sizes and a lack of external validation, leaving the generalizability of proposed models uncertain. In this study, we integrated multiphase CT radiomics, clinical parameters, and conventional morphological signs to develop and validate a combined prediction model. We further applied SHAP analysis to quantify feature contributions and enhance model transparency and clinical applicability (16).

Accordingly, we compared the diagnostic performance of unenhanced, arterial-phase, venous-phase, and multiphase CT radiomics models in a multicenter cohort, and constructed a combined nomogram to provide an intuitive, clinically actionable tool for preoperative risk assessment of thyroid nodules.

## 2 Materials and methods

### 2.1 Patient selection

This retrospective multicenter study was approved by the ethics committees of Quzhou People’s Hospital (Approval No. 2025-101). The requirement for written informed consent was waived.

We retrieved electronic medical records from Quzhou People’s Hospital (center 1, January 2017 to July 2024) and Hwa Mei Hospital, University of Chinese Academy of Sciences (center 2, October 2023 to August 2024). At center 1, 3106 patients with surgically resected thyroid nodules who underwent contrast-enhanced thyroid CT within 2 weeks before resection were initially screened; at center 2, 638 patients were initially screened. The detailed patient-selection flowchart is presented in **Figure 1**. Eligible patients met the following criteria: 1) underwent preoperative contrast-enhanced thyroid CT examination with standard unenhanced, arterial, and venous phases; 2) underwent thyroid surgery with confirmed pathological type; 3) solitary thyroid nodule with the longest diameter of ≥ 5 mm. Exclusion criteria included: 1) multiple thyroid nodules (center 1: n = 1523; center 2: n = 168); 2) history of malignant tumors (center 1: n = 40; center 2: n = 12); 3) prior surgery, radiotherapy, chemotherapy, or fine-needle aspiration biopsy before CT examination (center 1: n = 741; center 2: n = 237); 4) severe artifacts or damage in CT images (center 1: n = 77; center 2: n = 13). Because only solitary nodules were enrolled, each patient contributed exactly one index nodule, and all cohort assignments were performed at the patient level. The flow of patient selection is shown in **Figure 1**.

**Figure 1:**
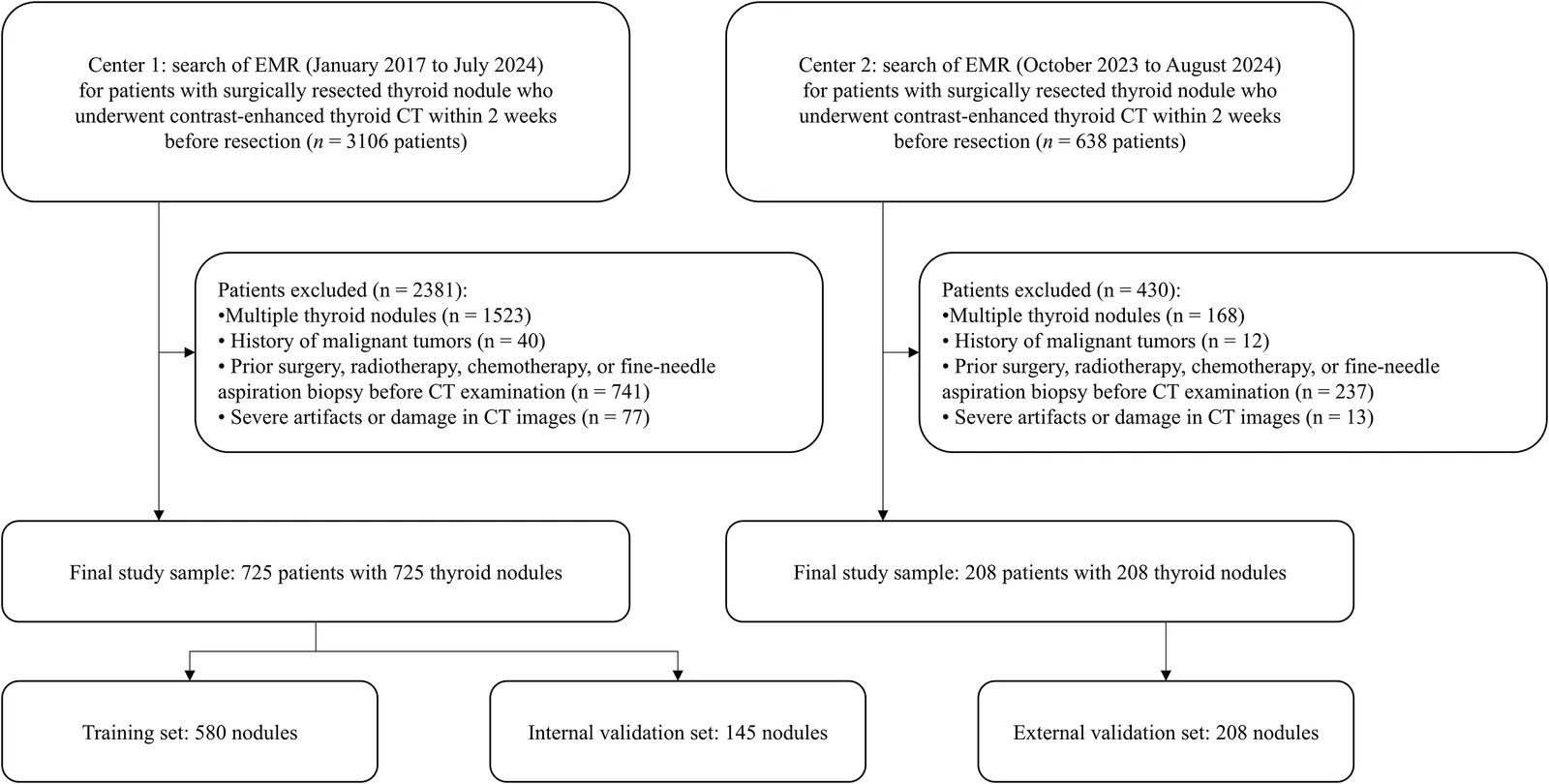
Flowchart of the patient selection.

After screening, 725 patients (417 benign, 308 malignant) from center 1 were randomly assigned at an 8:2 ratio to a training cohort (n = 580) and an internal validation cohort (n = 145). A total of 208 patients (58 benign, 150 malignant) from center 2 were included as a completely independent external validation cohort.

### 2.2 CT image acquisition

For center 1, all CT examinations were performed using a 320-detector row CT scanner (Aquilion ONE TSX-301C; Toshiba Medical Systems, Otawara, Japan). Scanning parameters were as follows: tube voltage, 100 kV; automatic tube current modulation; slice thickness/intervals, 0.5 mm; matrix, 512 × 512. A total of 70 mL of iopromide was intravenously injected at a rate of 3.0 mL/s, followed by a 20 mL saline flush. Arterial-phase and venous-phase images were acquired at 30 s and 60 s after contrast administration, respectively. The scanning range extended from the skull base to the level of the clavicles.

For center 2, all CT examinations were performed using a 64-slice spiral CT scanner (SOMATOM Definition; SIEMENS, Erlangen, Germany). Scanning parameters were as follows: tube voltage, 120 kV; tube current, 150–200 mAs; pitch, 1; rotation time, 1.0 s; detector collimation, 16 × 0.75 mm or 64 × 0.6 mm; field of view, 500 × 500 mm; matrix, 512 × 512; slice thickness, 5.0 mm. A total of 85 mL of iohexol (350 mg I/mL) was intravenously injected at a rate of 2.5–3.0 mL/s using a high-pressure syringe (SCT-210; MEDRAD). Arterial-phase and venous-phase images were acquired at 20 s and 35 s after contrast administration, respectively. The scanning range extended from the skull base to the level of the clavicles.

Prior to radiomics feature extraction, all images were resampled to an isotropic voxel size of 1 × 1 × 1 mm, with a unified window width of 250 HU and window level of 50 HU.

### 2.3 Assessment of CT morphological features and radiomics analysis

Two board-certified radiologists from center 1 independently reviewed all CT images, with knowledge of nodule location but blinded to other clinical and pathologic details. Four morphological features were evaluated: irregular shape, bite sign, microcalcification and range reduction. Discrepancies were resolved by consensus discussion.

Irregular shape: defined as a nodule that is not round or oval; otherwise, the shape was considered regular. Bite sign: defined as the nodule contacting the thyroid margin, with its maximum diameter located at or beyond the thyroid edge (17). Microcalcification: defined as calcifications with a maximum diameter ≤2.0 mm within the nodule. Range reduction: defined as a decrease in the density difference between the nodule and adjacent thyroid tissue on contrast-enhanced images compared with unenhanced images.

Radiologist 1 manually delineated the region of interest (ROI) slice by slice on all three phase images using ITK-SNAP software (version 4.2; www.itksnap.org) to generate a three-dimensional volume of interest (VOI) (18). To assess interobserver reproducibility of feature extraction, 30 cases were randomly selected for independent segmentation by Radiologist 2. The intraclass correlation coefficient (ICC) was calculated for each feature; only features with ICC > 0.75 were retained for subsequent analysis (**Supplementary Note 1**).

### 2.4 Model construction

For the clinical model, univariate analysis was first performed in the training cohort to compare variables including age, gender, nodule location, diameter, thyroid biochemical markers and CT morphological features. Variables with statistical significance on univariate analysis were entered into a multivariate logistic regression model to identify independent risk factors, which were subsequently used to construct the clinical model.

Feature selection involved a multi-step process. Initially, features with significant differences were identified using a t-test or U-test (p < 0.05). Subsequently, Pearson correlation analysis was applied to reduce redundancy by removing one feature from pairs with a correlation coefficient > 0.9. The minimum redundancy maximum relevance (mRMR) method was then used to select features with an optimal balance between relevance and redundancy. Finally, least absolute shrinkage and selection operator (LASSO) regression was employed for the final selection (**Supplementary Figure S1**). To minimize information leakage, all data-dependent preprocessing and feature-selection steps were conducted strictly within the training cohort, with the external validation cohort entirely excluded from these procedures as well as from subsequent model training and hyperparameter tuning. A multilayer perceptron (MLP) classifier was then used to build radiomics models for the unenhanced, arterial-phase, venous-phase, and multiphase CT datasets, respectively. The MLP was implemented with two hidden layers (128 and 64 neurons), ReLU activation, and a dropout rate of 0.5 after each hidden layer; it was trained with the Adam optimizer (learning rate = 0.001, batch size = 32) for a maximum of 500 epochs, with early stopping (patience = 20) based on internal validation loss. The multiphase model incorporated all informative radiomics features screened from the three CT phases to compare the diagnostic gain of multiphase versus single-phase information (**Supplementary Note 2**). The combined model integrated both independent clinical-morphological predictors and multiphase CT radiomics features to provide a comprehensive assessment of malignant risk. SHapley Additive exPlanations (SHAP) was employed to visualize and interpret feature contributions to the prediction model (**Figure 2**). Following the above-described workflow, a total of 1834 radiomic features were initially extracted from each of the three CT phases (unenhanced, arterial, and venous), yielding 5502 candidate features for the multiphase model. The detailed multi-step feature selection process is provided in **Supplementary Note 3**. Ultimately, 25, 19, and 35 non-zero-coefficient features were retained for the unenhanced, arterial, and venous phases, respectively, and 35 features were retained for the final multiphase radiomics signature.

**Figure 2:**
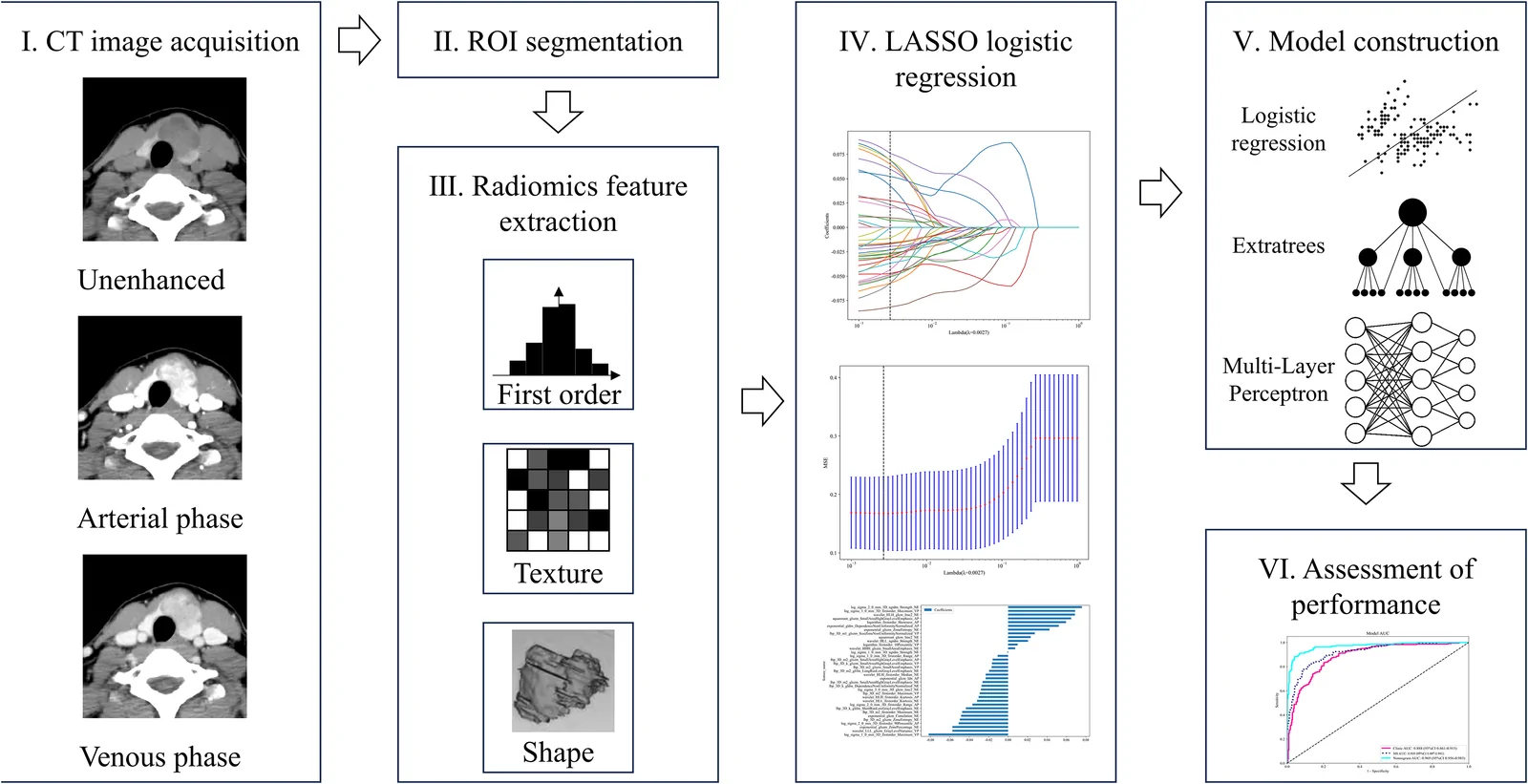
Flowchart of the radiomics analysis.

### 2.5 Statistical analysis

All statistical analyses were performed using R software (version 4.2.1; R Foundation for Statistical Computing, Vienna, Austria) and SPSS software (version 26.0; IBM Corp., Armonk, NY, USA). Continuous variables were expressed as mean ± standard deviation, and compared using the independent samples t-test or Mann–Whitney U test, as appropriate based on distribution. Categorical variables were presented as frequencies and percentages, and compared using the chi-square test or Fisher’s exact test. A comparative analysis of receiver operating characteristic curves was performed using the DeLong method.

Univariate and multivariate logistic regression analyses were performed to identify independent clinical predictors. Variables with p < 0.05 on univariate analysis and established clinical relevance were included in the multivariate model. A two-tailed p-value < 0.05 was considered statistically significant.

## 3 Results

### 3.1 Baseline characteristics

Baseline characteristics, including age, gender, nodule location, diameter, thyroid biochemical markers and CT morphological features, were compared across cohorts. No significant differences in major clinical and morphological variables were observed between the training and validation cohorts (**Table 1**).

**Table 1:** Patient baseline characteristics of the training, internal validation and external validation cohorts.

Characteristics	Training cohort		*P*-value	Internal validation cohort		*P*-value	External validation cohort		*P*-value
	Benign	Malignant		Benign	Malignant		Benign	Malignant	
Age	49.73 ± 11.68	42.72 ± 10.84	<0.001	49.72 ± 10.73	42.00 ± 10.33	<0.001	44.55 ± 12.65	42.15 ± 12.41	0.187
Diameter	2.52 ± 1.14	1.26 ± 0.84	<0.001	2.34 ± 1.05	1.20 ± 0.92	<0.001	2.57 ± 1.48	1.10 ± 0.75	<0.001
TPOAB	46.34 ± 236.71	89.89 ± 316.74	0.002	19.78 ± 98.40	39.93 ± 241.68	0.199	101.55 ± 242.29	182.12 ± 383.85	0.488
TGAB	63.48 ± 558.72	103.85 ± 529.97	<0.001	30.14 ± 116.75	158.45 ± 751.29	0.382	35.48 ± 156.08	19.17 ± 76.22	0.223
FT3	4.48 ± 0.62	4.44 ± 0.59	0.501	4.57 ± 0.74	4.55 ± 0.48	0.504	5.33 ± 0.68	5.32 ± 0.66	0.590
FT4	13.31 ± 2.08	13.25 ± 2.06	0.888	13.42 ± 2.00	12.70 ± 1.56	0.020	15.14 ± 1.99	15.33 ± 2.17	0.570
TSH	1.66 ± 1.18	1.90 ± 1.42	0.002	1.79 ± 1.12	2.00 ± 1.38	0.465	1.93 ± 1.31	2.02 ± 1.16	0.375
TT3	1.62 ± 0.33	1.59 ± 0.30	0.120	1.63 ± 0.34	1.55 ± 0.24	0.190	1.61 ± 0.17	1.62 ± 0.18	0.433
TT4	100.45 ± 18.48	97.30 ± 16.74	0.044	97.11 ± 18.15	94.91 ± 19.80	0.689	102.02 ± 10.60	102.30 ± 11.27	0.817
Gender			0.872			0.312			<0.001
Female	83(24.27%)	60(25.21%)		15(20.00%)	20(28.57%)		58(100.00%)	0(0.00%)	
Male	259(75.73%)	178(74.79%)		60(80.00%)	50(71.43%)		0(0.00%)	150(100.00%)	
Position			0.796			0.687			0.518
Right lobe	180(52.63%)	132(55.46%)		49(65.33%)	42(60.00%)		29(50.00%)	72(48.00%)	
Left lobe	151(44.15%)	99(41.60%)		23(30.67%)	26(37.14%)		28(48.28%)	70(46.67%)	
Isthmus	11(3.22%)	7(2.94%)		3(4.00%)	2(2.86%)		1(1.72%)	8(5.33%)	
Microcalcification			0.002			1.000			0.248
Negative	316(92.40%)	200(84.03%)		67(89.33%)	62(88.57%)		53(91.38%)	126(84.00%)	
Positive	26(7.60%)	38(15.97%)		8(10.67%)	8(11.43%)		5(8.62%)	24(16.00%)	
Irregular shape			<0.001			<0.001			0.024
Negative	280(81.87%)	57(23.95%)		63(84.00%)	16(22.86%)		34(58.62%)	60(40.00%)	
Positive	62(18.13%)	181(76.05%)		12(16.00%)	54(77.14%)		24(41.38%)	90(60.00%)	
Bite sign			1.000			0.435			0.289
Negative	25(7.31%)	17(7.14%)		8(10.67%)	4(5.71%)		4(6.90%)	20(13.33%)	
Positive	317(92.69%)	221(92.86%)		67(89.33%)	66(94.29%)		54(93.10%)	130(86.67%)	
Range reduction			<0.001			<0.001			0.006
Negative	277(80.99%)	75(31.51%)		58(77.33%)	19(27.14%)		50(86.21%)	99(66.00%)	
Positive	65(19.01%)	163(68.49%)		17(22.67%)	51(72.86%)		8(13.79%)	51(34.00%)	

Baseline characteristics of the enrolled patients data are presented as median ± standard deviation or n (%). P values are for comparisons between the benign and malignant nodule groups.

Multivariate logistic regression identified seven independent predictors of malignant thyroid nodules: diameter, age, total thyroxine (TT4), free thyroxine (FT4), bite sign, irregular shape and range reduction (**Table 2**) (**Supplementary Table S1**).

**Table 2:** Univariable and multivariable logistic regression analysis for the association with surgical risk.

Characteristic	Univariate logistic regression		Multivariate logistic regression	
	*OR (95% CI)*	*p-value*	*OR (95% CI)*	*p-value*
Diameter	0.661(0.614-0.710)	<0.001	0.180(0.133-0.245)	<0.001
Bite sign	0.697(0.604-0.805)	<0.001	5.526(2.241-13.626)	0.002
FT4	0.973(0.964-0.983)	<0.001	1.224(1.082-1.385)	0.007
Age	0.990(0.987-0.993)	<0.001	0.964(0.946-0.983)	0.002
TT4	0.996(0.995-0.998)	<0.001	0.969(0.952-0.986)	0.004
Range reduction	2.508(1.970-3.193)	<0.001	4.443(2.635-7.493)	<0.001
Irregular shape	2.919(2.291-3.717)	<0.001	41.612(22.737-76.172)	<0.001

CI, confidence interval.

### 3.2 Performance of prediction models

The clinical model was constructed using the seven aforementioned clinical and morphological predictors. It achieved an AUC of 0.800 (95% CI: 0.732–0.868) in the external validation cohort. At the optimal cutoff value derived from the training cohort, it yielded a sensitivity of 78.0%, specificity of 72.4%, and accuracy of 76.4%, indicating moderate discriminatory ability based on clinical and morphological features alone (**Table 3**).

**Table 3:** Receiver operating characteristic curves of the nomogram, Multi-sequence radiomics and clinical models.

Model	Accuracy	AUC	95% CI	Sensitivity	Specificity	PPV	NPV	Precision	Recall	F1	Threshold
Training cohort											
Clinical Model	0.812	0.888	0.861 - 0.915	0.832	0.798	0.742	0.872	0.742	0.832	0.784	0.198
MS Model	0.853	0.919	0.897 - 0.941	0.824	0.874	0.820	0.877	0.820	0.824	0.822	0.411
Nomogram	0.922	0.969	0.956 - 0.983	0.912	0.930	0.900	0.938	0.900	0.912	0.906	0.444
Internal validation cohort											
Clinical Model	0.779	0.833	0.765 - 0.901	0.814	0.747	0.750	0.812	0.750	0.814	0.781	0.201
MS Model	0.793	0.836	0.770 - 0.901	0.800	0.787	0.778	0.808	0.778	0.800	0.789	0.277
Nomogram	0.890	0.945	0.912 - 0.978	0.857	0.920	0.909	0.873	0.909	0.857	0.882	0.531
External validation cohort											
Clinical Model	0.764	0.800	0.732 - 0.868	0.780	0.724	0.880	0.560	0.880	0.780	0.827	0.647
MS Model	0.808	0.787	0.708 - 0.866	0.840	0.724	0.887	0.636	0.887	0.840	0.863	0.157
Nomogram	0.769	0.865	0.812 - 0.918	0.740	0.845	0.925	0.557	0.925	0.740	0.822	0.464

MS Model, Multi-sequence radiomics model; AUC, area under the receiver operating characteristic curve; CI, confidence interval; PPV, positive predictive value (proportion of true positives among predicted positives); NPV, negative predictive value (proportion of true negatives among predicted negatives); F1, harmonic mean of precision and sensitivity, balancing both metrics.

Among the single-phase radiomics models, the venous-phase model demonstrated the best performance, with an external validation AUC of 0.815 (95% CI: 0.745–0.885), sensitivity of 88.0%, specificity of 67.2%, and accuracy of 82.2% (**Table 3**). The external validation AUCs of the unenhanced, arterial-phase, and multiphase models were 0.785, 0.785, and 0.787, respectively (**Table 4**). While the multiphase model showed superior performance in the training and internal validation cohorts, the venous-phase model exhibited more consistent performance in the external validation, highlighting the prominent diagnostic value of venous-phase features for differentiating benign and malignant nodules.

**Table 4:** Comparison of different single-phase models and multi-sequence radiomics model.

Model	Accuracy	AUC	95% CI	Sensitivity	Specificity	PPV	NPV	Precision	Recall	F1	Threshold
Training cohort											
NE Model	0.828	0.880	0.851 - 0.908	0.798	0.848	0.785	0.858	0.785	0.798	0.792	0.425
AP Model	0.843	0.914	0.892 - 0.937	0.870	0.825	0.775	0.901	0.775	0.870	0.820	0.360
VP Model	0.803	0.885	0.857 - 0.913	0.794	0.810	0.744	0.850	0.744	0.794	0.768	0.352
MS Model	0.853	0.919	0.897 - 0.941	0.824	0.874	0.820	0.877	0.820	0.824	0.822	0.411
Internal validation cohort											
NE Model	0.793	0.811	0.736 - 0.886	0.871	0.720	0.744	0.857	0.744	0.871	0.803	0.226
AP Model	0.793	0.812	0.741 - 0.883	0.829	0.760	0.763	0.826	0.763	0.829	0.795	0.322
VP Model	0.807	0.835	0.767 - 0.902	0.886	0.733	0.756	0.873	0.756	0.886	0.816	0.229
MS Model	0.793	0.836	0.770 - 0.901	0.800	0.787	0.778	0.808	0.778	0.800	0.789	0.277
External validation cohort											
NE Model	0.788	0.785	0.706 - 0.864	0.807	0.741	0.890	0.597	0.890	0.807	0.846	0.295
AP Model	0.798	0.785	0.706 - 0.864	0.827	0.724	0.886	0.618	0.886	0.827	0.855	0.216
VP Model	0.822	0.815	0.745 - 0.885	0.880	0.672	0.874	0.684	0.874	0.880	0.877	0.127
** MS Model**	**0.808**	**0.787**	**0.708 - 0.866**	**0.840**	**0.724**	**0.887**	**0.636**	**0.887**	**0.840**	**0.863**	**0.157**

NE Model, Unenhanced radiomics model; AP Model, Arterial phase radiomics model; VP Model, Venous phase radiomics model; MS Model, Multi-sequence radiomics model; AUC, area under the receiver operating characteristic curve; CI, confidence interval; PPV, positive predictive value (proportion of true positives among predicted positives); NPV, negative predictive value (proportion of true negatives among predicted negatives); F1, harmonic mean of precision and sensitivity, balancing both metrics. Bold values indicate the best (optimal) performance metrics among the compared models within each cohort (training, internal validation, and external validation).

The combined model, which integrated clinical and morphological features and multiphase radiomics features, achieved the highest overall diagnostic performance, with an external validation AUC of 0.865 (95% CI: 0.812–0.918), sensitivity of 74.0%, specificity of 84.5%, and accuracy of 76.9% (**Table 3**). A radiomics nomogram was subsequently established based on this combined model (**Figure 3**).

**Figure 3:**
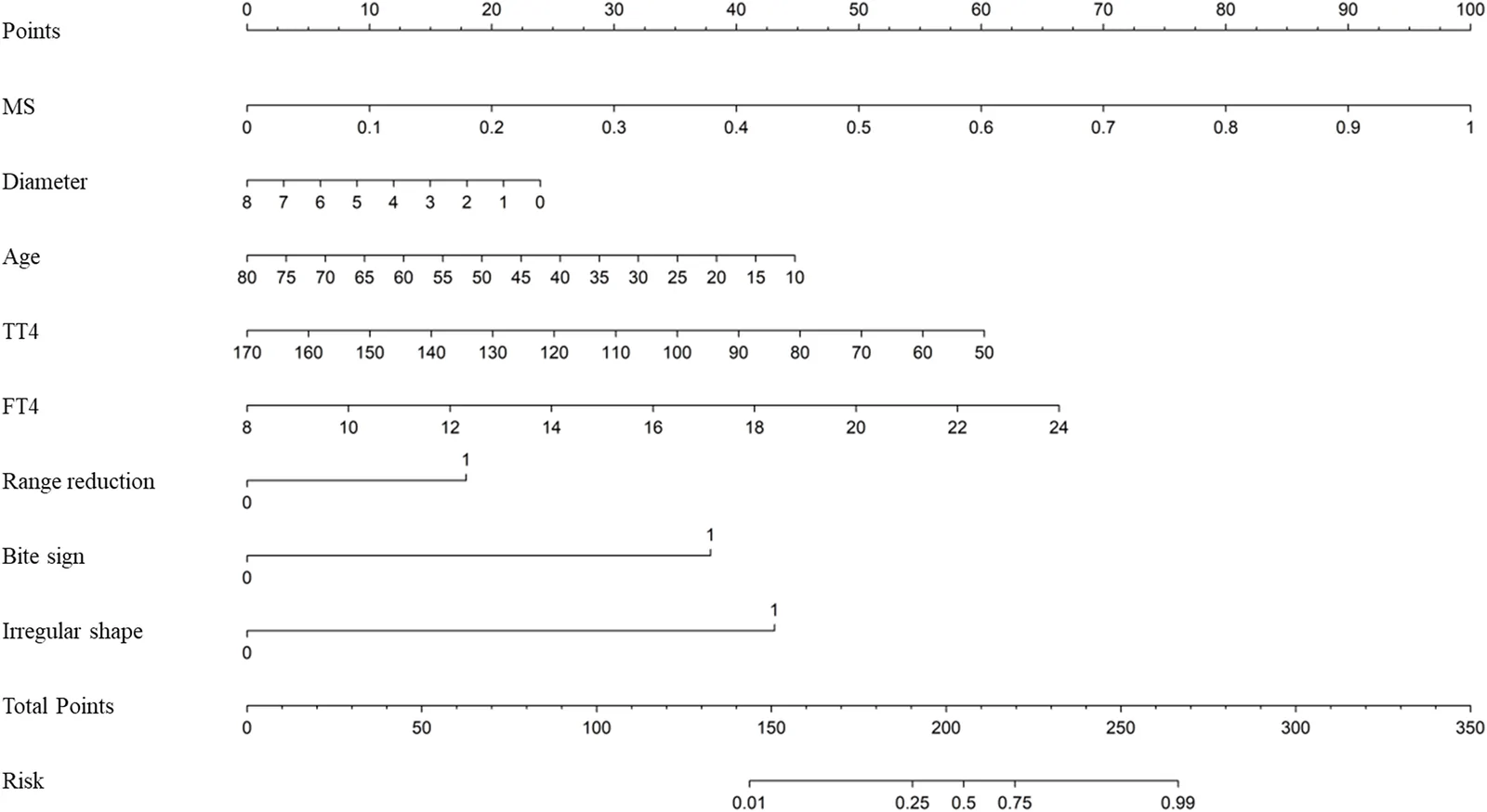
Radiomics nomogram for the prediction of benign and malignant thyroid nodules.

### 3.3 Model comparison in the external validation cohort

In the external validation cohort, the combined model had a significantly higher AUC than both the clinical model and the multiphase radiomics model. **Figure 4** shows its ROC curve in all three models, while the results of the DeLong test comparing these models’ performances are displayed in **Supplementary Figure S2**.

**Figure 4:**
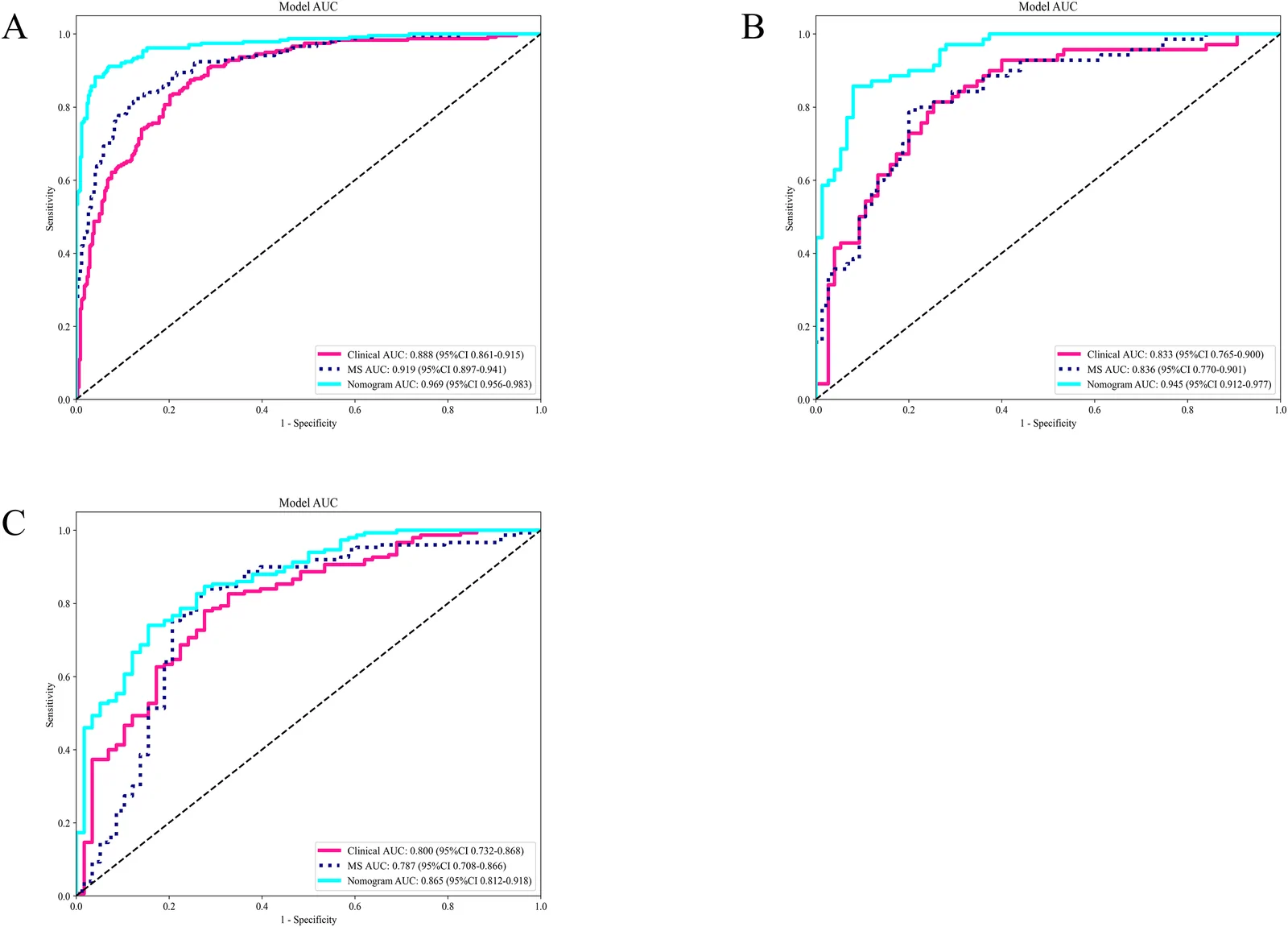
ROC curves. ROC curves show sensitivity and specificity of different models in training **(A)**, internal validation **(B)**, and external validation **(C)** sets. Data in parentheses are 95% CIs.

Calibration curve analysis demonstrated that the combined model had the lowest Brier score, indicating closer agreement between predicted probabilities and actual malignant risk (**Supplementary Figure S3**). Decision curve analysis revealed that the combined model yielded a higher net benefit across most threshold probability ranges, supporting its superior clinical utility for auxiliary decision-making (**Supplementary Figure S4**).

### 3.4 Model interpretability

To visually interpret the selected variables, we applied SHAP to illustrate how these variables contributed to the prediction of benign and malignant thyroid nodules in the model.

A SHAP beeswarm plot is shown in **Figure 5**, which provides a global view of how the value of each feature impacts the model prediction for all the samples. Dot colors denote feature magnitudes (red for high values, blue for low), while x-axis SHAP values reflect predictive effects: positive values raise the positive-class probability and negative values reduce it. Features are vertically ranked by mean absolute SHAP values to quantify their overall predictive importance.

**Figure 5:**
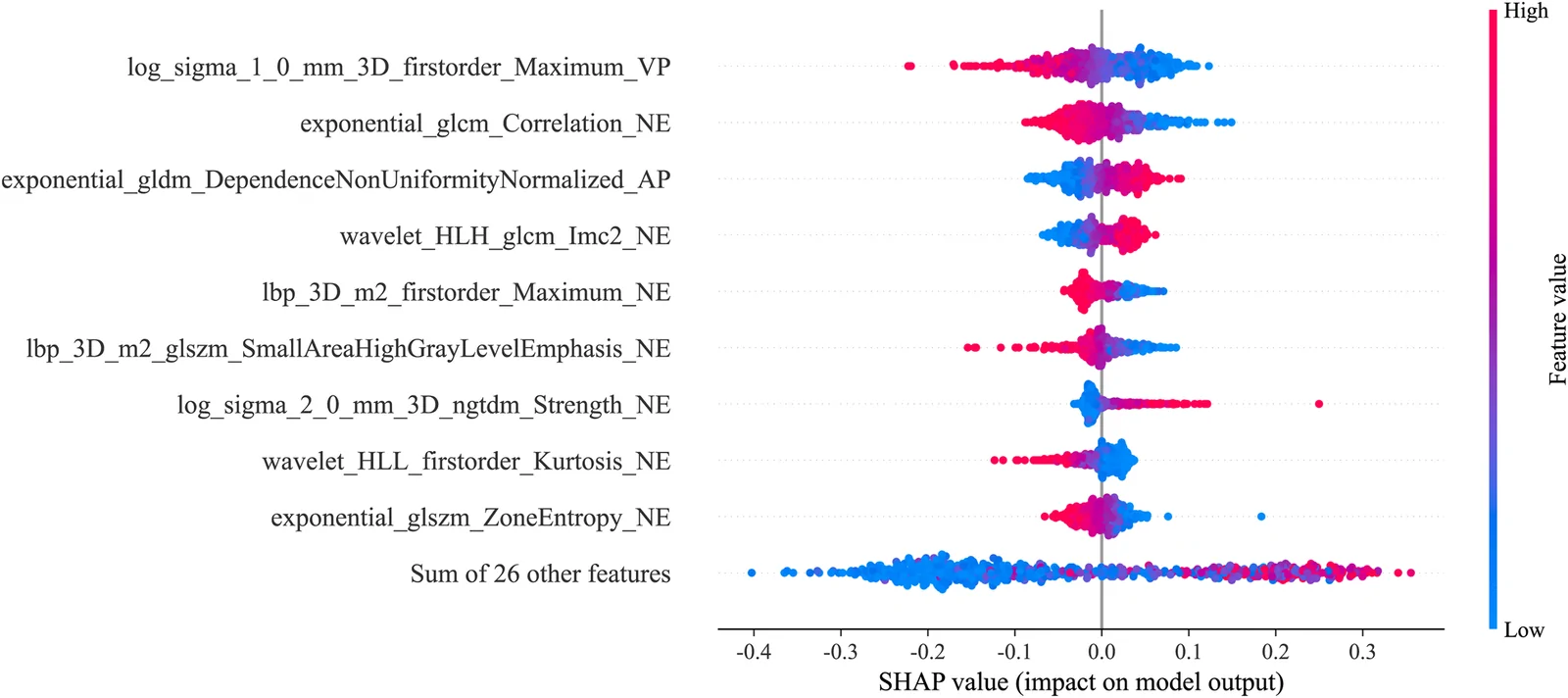
SHAP beeswarm plot. SHAP beeswarm plot of the nine most important features (ranked by mean |SHAP|). The horizontal axis represents SHAP value, indicating a feature’s contribution to the model output (SHAP > 0 favors the “positive” class, SHAP < 0 favors the “negative” class). Color indicates the original feature value (red: high, blue: low). Feature naming: ‘_NE’, Unenhanced; ‘_AP’, Arterial phase; ‘_VP’, Venous phase. SHAP, SHapley Additive exPlanations.

To elucidate feature contributions at the individual level, two representative patients were analyzed via SHAP waterfall and force plots (**Figure 6**). Patient A, pathologically confirmed as a benign thyroid nodule (true negative), obtained an accurate low-risk prediction with a SHAP value of 0.051 (**Figures 6A, B**), whereas Patient B with a pathologically proven malignant thyroid nodule (true positive) was correctly predicted to have high malignancy risk, corresponding to a SHAP value of 0.961 (**Figures 6C, D**).

**Figure 6:**
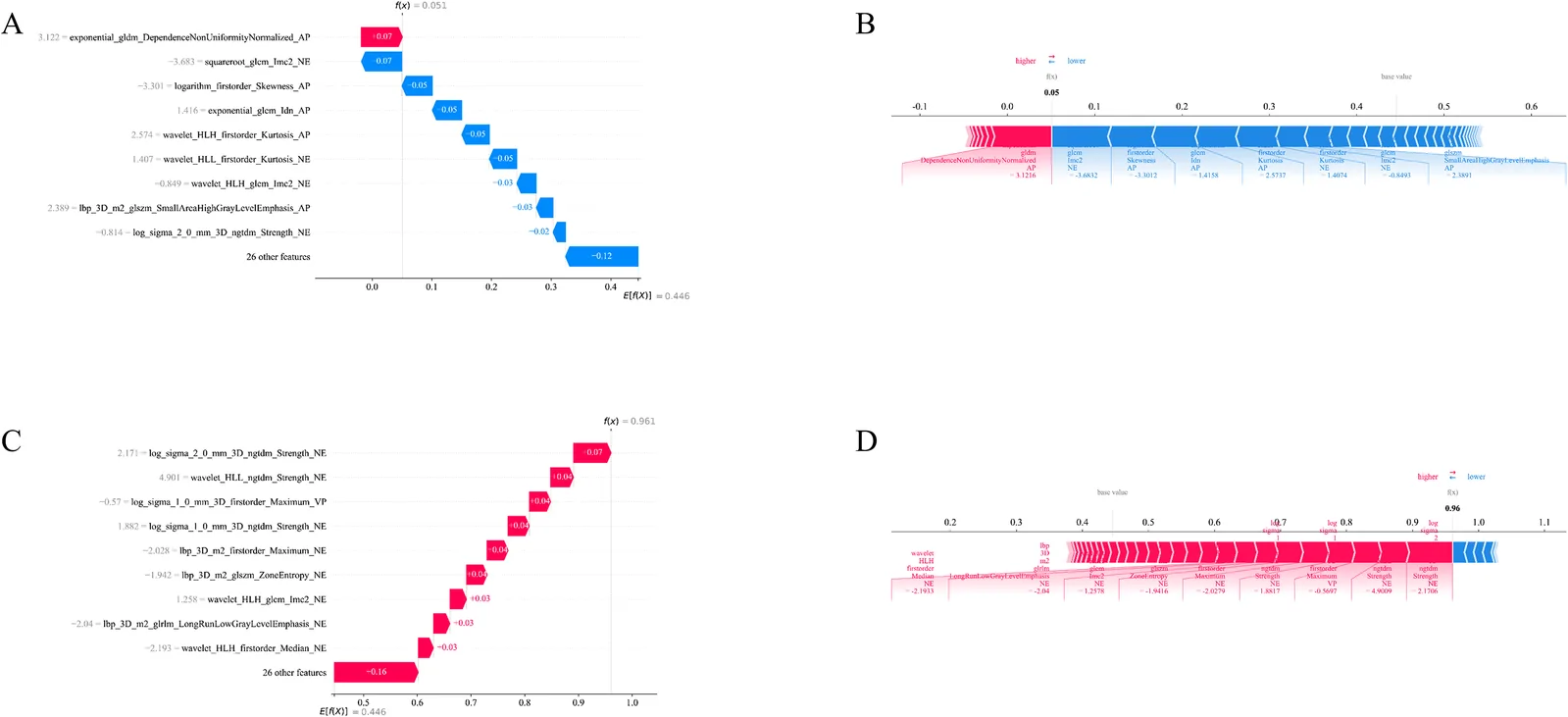
Personalized interpretation of the model by SHAP. Figure panels **(A, B)** show the SHAP waterfall and force plots for Patient 1 with pathologically confirmed benign thyroid nodule (true negative), whereas panels **(C, D)** display corresponding plots for Patient 2 diagnosed with malignant thyroid nodule (true positive). In waterfall plots, red bars stand for features promoting malignant risk, and blue bars denote risk-suppressing effects, with bar lengths proportional to contribution magnitude. f(X) refers to the model’s individualized predicted probability, while E[f(X)] denotes the baseline prediction value without feature input. Force plots visualize the cumulative additive impact of each feature, which shifts the baseline score to the final predictive result. Across all graphs, the base value equals the average model output; SHAP values quantify both the direction and magnitude of each feature’s predictive influence. Blue corresponds to reduced malignancy risk, and red to elevated malignancy risk. Feature naming: ‘_NE’, Unenhanced; ‘_AP’, Arterial phase; ‘_VP’, Venous phase. SHAP, SHapley Additive exPlanations.

Both predictions were concordant with postoperative histopathology, with clear directional contributions from individual features, demonstrating that the model can provide transparent, case-specific diagnostic rationale.

## 4 Discussion

In this multicenter study, we developed and validated a series of prediction models—including clinical model, single-phase and multiphase CT radiomics models, and combined model—for the preoperative differentiation of benign and malignant thyroid nodules using multiphase CT images and clinical data. The combined model achieved an AUC of 0.865 in the external validation cohort, significantly outperforming both the clinical model and the multiphase radiomics model. Calibration and decision curve analyses further confirmed its superior predictive consistency and clinical net benefit. These findings indicate that radiomics captures quantitative textural and heterogeneity information that is imperceptible to visual inspection, and that its integration with conventional clinical and morphological data can enhance preoperative risk stratification. The inclusion of an external validation cohort also strengthens the generalizability of our findings.

The clinical model, incorporating age, diameter, TT4, FT4, and CT morphological features (irregular shape, bite sign and range reduction), achieved an external validation AUC of 0.800, demonstrating fair discriminatory capacity. Notably, although microcalcification was evaluated as a candidate morphological feature, univariable logistic regression revealed that it did not reach statistical significance in our cohort (OR = 1.462, 95% CI: 0.962–2.221, p = 0.136; **Supplementary Table S1**); accordingly, it was not entered into the multivariable model per the prespecified variable-selection criterion (p < 0.05). The nonsignificant association may reflect limited statistical power owing to the restricted sample size and the relatively small number of participating centers, which could have introduced selection bias. Future multicenter studies with larger sample sizes and standardized CT imaging protocols are warranted to further clarify the independent predictive value of microcalcification in CT-based risk stratification of thyroid nodules. Among the morphological features retained in the final model, irregular shape, bite sign and range reduction are underpinned by pathological mechanisms including infiltrative tumor growth, capsular invasion, multifocal coalescence, and peritumoral fibrosis (19–21), all of which alter the interface between the nodule and surrounding normal thyroid parenchyma. However, this model remains limited by selection bias, the subjectivity of morphological interpretation, and interobserver variability. Large benign nodules may also abut or distort the thyroid capsule (22), and the underrepresentation of small benign nodules in our surgical cohort may further compromise the stability of a purely clinical model.

CT radiomics extracts quantitative grayscale, textural, and spatial heterogeneity features beyond the resolution of human visual assessment, and has been widely applied across multiple oncological domains. Liu et al. applied CT-based delta-radiomics analysis to preoperative prediction of ISUP pathological nuclear grading in clear cell renal cell carcinoma, with the delta-radiomics model achieving an AUC of 0.911 in the training cohort (23). Chen et al. proposed a spatially aware radiomics model integrating anatomical knowledge for lymph node malignancy prediction in head and neck cancer, which achieved an AUC of 0.953 and significantly outperformed the conventional radiomics model (AUC = 0.931) (24). Miao et al. developed a radiomics nomogram integrated with radiological features for preoperative prediction of lung nodule invasiveness, achieving a training AUC of 0.91 and substantially outperforming the Lung-RADS system (AUC = 0.669) (25). These cross-tumor successes provide a strong rationale for applying CT radiomics to thyroid cancer, where it has demonstrated value across several clinical tasks. Yu et al. constructed a multicenter CT-based radiomics nomogram for predicting extrathyroidal extension (ETE), yielding AUCs of 0.750 and 0.797 in the internal and external test sets, respectively (26). Xu et al. employed dual-energy CT (DECT) iodine map–based radiomics for ETE prediction and recurrence stratification, with the nomogram achieving AUCs of 0.78 and 0.84 in the training and validation cohorts (27). Zhou et al. applied DECT-derived iodine map radiomics to diagnose metastatic cervical lymph nodes, attaining a validation AUC of 0.895 and significantly outperforming qualitative CT evaluation (AUC = 0.728) (28). Mou et al. established a CT radiomics nomogram for central lymph node metastasis prediction in papillary thyroid microcarcinoma, achieving AUCs of 0.800 and 0.803 in the internal and external test sets and outperforming radiologists’ interpretation (AUC = 0.655) (29). Du et al. built a clinical-CT radiomic combined model for lateral cervical lymph node metastasis, with AUCs of 0.876 and 0.821 in the internal and external validation sets (30). Zhou et al. developed a multimodal vascular phenotype model integrating Doppler ultrasound and DECT for recurrence risk stratification, achieving an AUC of 0.815 for discriminating CD31 expression, where high CD31 expression significantly correlated with recurrence (p < 0.001) (31). Collectively, these studies underscore the broad clinical utility of CT radiomics in oncological imaging. In the present study, we compared unenhanced, arterial-phase, venous-phase, and multiphase radiomics models, and found that the venous-phase model outperformed all other single-phase models in external validation (AUC = 0.815), even exceeding the multiphase model. We postulate that venous-phase images more reliably reflect differences in stromal enhancement, contrast agent retention, and fibrotic content within lesions, while mitigating the influence of transient perfusion fluctuations inherent to arterial-phase imaging. This may explain why venous-phase features are more robust for distinguishing benign from malignant nodules.

Previous studies have reached inconsistent conclusions regarding the optimal CT phase for thyroid nodule characterization. Kong et al. reported superior performance for the arterial phase, which may be attributed to their early triggered scanning protocol, which captures the hypervascular profile of malignant nodules (32). In a study by Lin et al., non-uniform contrast enhancement protocols across centers compromised the cross-center stability of venous-phase features, leading to better external validation performance for the unenhanced model (18). Although the CT acquisition protocols differed between the two centers, the model maintained acceptable performance in the independent external cohort, suggesting a certain degree of robustness to protocol heterogeneity. Furthermore, standardized image preprocessing was performed prior to feature extraction to minimize equipment-related differences, which likely contributed to the robust generalizability of venous-phase features in our external validation cohort.

The combined model consistently achieved the best overall performance across all cohorts, indicating that the integration of clinical and morphological signs and radiomics features can mitigate the subjectivity of visual assessment and address the limitations of single-modality models. Compared with prior investigations, our study included a relatively large sample size and a dedicated external validation cohort, enhancing the reliability of our results. However, we did not include several clinically important CT features, such as cystic change and abnormal cervical lymph nodes, which may partially explain why our external validation AUC was slightly lower than that reported in some previous studies (18, 33–35). Further standardization of morphological feature assessment and inclusion of additional clinical variables may yield additional improvements to the combined model.

SHAP analysis provided additional insights into model interpretability. Features such as log_sigma_2_0_mm_3D_ngtdm_Strength_NE and wavelet_HLH_glcm_Imc2_NE reflect local grayscale heterogeneity and inter-voxel textural correlation, which likely correspond to intralesional structural complexity and tumor heterogeneity. Benign nodule-associated features were predominantly first-order statistics and markers of textural uniformity, whereas malignant nodule-associated features were more closely linked to multiscale grayscale inhomogeneity and textural complexity. At the individual patient level, benign cases were correctly stratified as low risk and malignant cases as high risk, in agreement with histopathology, demonstrating that this approach can provide visualized, personalized diagnostic evidence for clinical practice.

Our findings do not imply that CT radiomics replaces the foundational role of ultrasound and the ACR TI-RADS classification system in thyroid nodule evaluation. Rather, CT radiomics serves as a valuable adjunct for patients with incidentally detected thyroid nodules on CT, indeterminate ultrasound findings, a need for detailed assessment of adjacent anatomical structures, or those undergoing comprehensive preoperative risk stratification. It can inform decisions regarding further ultrasound evaluation, FNAB, or surgical management, and may help reduce unnecessary invasive procedures.

Several limitations of this study should be acknowledged. First, the retrospective design and exclusive inclusion of surgical patients may introduce selection bias. Second, we only included solitary nodules ≥ 5 mm in diameter, so our findings may not be generalizable to multiple nodules or nodules < 5 mm in diameter. Third, manual VOI segmentation is labor-intensive and may introduce interobserver variability; semi-automated or fully automated segmentation methods could improve efficiency and reproducibility in future work (36, 37). Finally, we did not perform head-to-head comparisons with ultrasound, ultrasound-based radiomics, or radiologists’ overall diagnostic impression. Future multicenter prospective studies are warranted to develop more robust multimodal models, incorporating automated segmentation, deep learning, ultrasound features, and molecular biomarkers.

In conclusion, venous-phase CT radiomics features exhibit robust diagnostic value for differentiating benign and malignant thyroid nodules. Integration with clinical and morphological information further improves diagnostic accuracy, stability, and clinical utility, providing a novel quantitative tool for the preoperative management of incidentally detected thyroid nodules on CT.

## 5 Conclusion

The combined model yielded the best diagnostic performance, superior to both the clinical model and the multiphase radiomics model. The venous-phase radiomics model showed the most robust cross-center generalizability among all single-phase models.

Supported by multicenter external validation and SHAP-based interpretability, this combined model may provide a noninvasive, objective reference for risk stratification of incidentally detected thyroid nodules on CT, aiding clinical decision-making and informing future multimodal investigations.

## Data Availability

The raw data supporting the conclusions of this article will be made available by the authors, without undue reservation.
