# Supplementary material for: Computed tomography multi-phase radiomics models for differentiating benign and malignant thyroid nodules
Source: Front Endocrinol (Lausanne). 2026 Oct 15;17:1928263. doi: 10.3389/fendo.2026.1928263 (PMC13716650; doi:10.3389/fendo.2026.1928263)
Supplement: Supplementary file 1 [file DataSheet2.docx]

Radiomics features for Multi-sequence radiomics model construction:

Rad_signature = 0.4103448275862061 + +0.068816 * wavelet_HLH_glcm_Imc2_NE -0.057329 * wavelet_LLL_glszm_GrayLevelVariance_VP -0.030361 * wavelet_HLH_firstorder_Kurtosis_AP -0.029254 * lbp_3D_m2_firstorder_Maximum_VP +0.075946 * log_sigma_2_0_mm_3D_ngtdm_Strength_NE -0.081753 * log_sigma_1_0_mm_3D_firstorder_Maximum_VP -0.049667 * lbp_3D_m2_glszm_ZoneEntropy_NE -0.048445 * exponential_glcm_Correlation_NE +0.069046 * log_sigma_3_0_mm_3D_firstorder_Maximum_VP +0.052252 * exponential_gldm_DependenceNonUniformityNormalized_AP -0.027959 * log_sigma_3_0_mm_3D_glcm_Imc2_NE -0.027392 * lbp_3D_k_gldm_DependenceNonUniformityNormalized_NE -0.047291 * lbp_3D_m2_firstorder_Maximum_NE -0.017028 * lbp_3D_m2_glszm_SmallAreaEmphasis_VP +0.020503 * wavelet_HLL_ngtdm_Strength_NE +0.023254 * squareroot_glcm_Imc2_NE -0.023070 * wavelet_HLH_firstorder_Median_NE +0.042569 * exponential_glszm_ZoneEntropy_NE +0.064843 * squareroot_glszm_SmallAreaHighGrayLevelEmphasis_AP +0.007298 * wavelet_HHH_glszm_SmallAreaEmphasis_NE -0.031981 * wavelet_HLL_firstorder_Kurtosis_NE -0.019568 * lbp_3D_m2_glrlm_LongRunLowGrayLevelEmphasis_NE -0.016605 * lbp_3D_k_glszm_SmallAreaHighGrayLevelEmphasis_VP -0.043402 * lbp_3D_k_glrlm_ShortRunLowGrayLevelEmphasis_NE -0.026244 * lbp_3D_m2_glszm_SmallAreaHighGrayLevelEmphasis_NE -0.036706 * log_sigma_2_0_mm_3D_firstorder_Range_AP -0.023134 * exponential_glcm_Idn_AP -0.015896 * lbp_3D_m2_glszm_SmallAreaHighGrayLevelEmphasis_AP -0.050880 * log_sigma_2_0_mm_3D_firstorder_90Percentile_AP +0.027484 * lbp_3D_m1_glszm_SizeZoneNonUniformityNormalized_VP +0.059662 * logarithm_firstorder_Skewness_AP +0.009802 * logarithm_firstorder_10Percentile_VP -0.057278 * exponential_glszm_ZonePercentage_NE -0.010722 * log_sigma_1_0_mm_3D_firstorder_Range_AP -0.000582 * log_sigma_1_0_mm_3D_ngtdm_Strength_NE
