# Supplementary material for: Computed tomography multi-phase radiomics models for differentiating benign and malignant thyroid nodules
Source: Front Endocrinol (Lausanne). 2026 Oct 15;17:1928263. doi: 10.3389/fendo.2026.1928263 (PMC13716650; doi:10.3389/fendo.2026.1928263)
Supplement: Supplementary file 2 [file DataSheet3.docx]

Radiomics Feature Selection

Unenhanced Phase

A total of 1,834 radiomic features were initially extracted. First, Z-score standardization was performed for each radiomic feature, after which t-test filtering retained 1,603 radiomic features. Subsequent Pearson correlation coefficient analysis combined with minimum redundancy maximum relevance (mRMR) selection further reduced the feature set to 40 features. Finally, least absolute shrinkage and selection operator (LASSO) regression retained 25 radiomic features for model construction.

Arterial Phase

A total of 1,834 radiomic features were initially extracted. First, Z-score standardization was performed for each radiomic feature, after which t-test filtering retained 1,594 radiomic features. Subsequent Pearson correlation coefficient analysis combined with mRMR selection further reduced the feature set to 40 features. Finally, LASSO regression retained 19 radiomic features for model construction.

Venous Phase

A total of 1,834 radiomic features were initially extracted. First, Z-score standardization was performed for each radiomic feature, after which t-test filtering retained 1,588 radiomic features. Subsequent Pearson correlation coefficient analysis combined with mRMR selection further reduced the feature set to 40 features. Finally, LASSO regression retained 35 radiomic features for model construction.

Multiphase Combined Model

A total of 5,502 radiomic features were initially extracted. First, Z-score standardization was performed for each radiomic feature, after which t-test filtering retained 4,755 radiomic features. Subsequent Pearson correlation coefficient analysis combined with mRMR selection further reduced the feature set to 40 features. Finally, LASSO regression retained 35 radiomic features for model construction.
