# Supplementary material for: Computed tomography multi-phase radiomics models for differentiating benign and malignant thyroid nodules
Source: Front Endocrinol (Lausanne). 2026 Oct 15;17:1928263. doi: 10.3389/fendo.2026.1928263 (PMC13716650; doi:10.3389/fendo.2026.1928263)
Supplement: Supplementary file 3 [file DataSheet4.docx]

Figure S1 Radiomics feature selection

The figure illustrates the process of radiomics feature selection. A. The feature selection of LASSO regression is based on the minimum binomial deviation of the model plus one standard error to review features with non-zero coefficients. B. The 5-fold cross-validation process for LASSO regression feature selection is based on the minimum binomial deviation of the model plus one standard error, multiplied by the logarithm of λ. This value is equal to 0.0110. C. Proving the importance of specific radiomics features through histograms

Figure S2 DeLong test

The P-value of DeLong test between different models in training (A), internal validation (B), and external validation (C) sets.

Figure S3 Calibration curves

Calibration curves show actual probability and predicted probability of different models in training (A), internal validation (B), and external validation (C) sets

Figure S4 Decision curves

Decision curves show standardized net benefit and high-risk threshold of different models in training (A), internal validation (B), and external validation (C) sets.
