# Supplementary material for: Computed tomography multi-phase radiomics models for differentiating benign and malignant thyroid nodules
Source: Front Endocrinol (Lausanne). 2026 Oct 15;17:1928263. doi: 10.3389/fendo.2026.1928263 (PMC13716650; doi:10.3389/fendo.2026.1928263)
Supplement: Supplementary file 4 [file Table1.docx]

**Table S1** Univariable and multivariable Logistic Regression Analysis for the Association with Surgical Risk

| **Characteristic** | **Univariate Logistic Regression** |  |  | **Multivariate Logistic Regression** |  |
| --- | --- | --- | --- | --- | --- |
|  | ***OR (95% CI)*** | ***p_value*** |  | ***OR (95% CI)*** | ***p_value*** |
| Diameter | 0.661(0.614-0.710) | <0.001 |  | 0.180(0.133-0.245) | <0.001 |
| Gender | 0.687(0.586-0.807) | <0.001 |  | 0.758(0.428-1.343) | 0.425 |
| Bite sign | 0.697(0.604-0.805) | <0.001 |  | 5.526(2.241-13.626) | 0.002 |
| TT3 | 0.794(0.729-0.865) | <0.001 |  | 0.968(0.278-3.367) | 0.965 |
| Position | 0.798(0.731-0.871) | <0.001 |  | 1.030(0.663-1.600) | 0.911 |
| TSH | 0.917(0.859-0.978) | 0.027 |  | 0.989(0.807-1.213) | 0.930 |
| FT3 | 0.922(0.893-0.950) | <0.001 |  | 1.376(0.729-0.865) | 0.315 |
| FT4 | 0.973(0.964-0.983) | <0.001 |  | 1.224(0.816-2.319) | 0.007 |
| Age | 0.990(0.987-0.993) | <0.001 |  | 0.964(0.946-0.983) | 0.002 |
| TT4 | 0.996(0.995-0.998) | <0.001 |  | 0.969(0.952-0.986) | 0.004 |
| TPOAB | 1.000(1.000-1.001) | 0.415 |  |  |  |
| TGAB | 1.000(1.000-1.000) | 0.822 |  |  |  |
| Microcalcification | 1.462(0.962-2.221) | 0.136 |  |  |  |
| Range reduction | 2.508(1.970-3.193) | <0.001 |  | 4.443(2.635-7.493) | <0.001 |
| Irregular shape | 2.919(2.291-3.717) | <0.001 |  | 41.612(22.737-76.172) | <0.001 |

**Notes**: CI: confidence interval;
